# Assessing the Association Between Lead Pollution and Risk of Alzheimer’s Disease by Integrating Multigenomics

**DOI:** 10.3389/fnins.2022.880105

**Published:** 2022-07-22

**Authors:** Chunying Li, Yuwei Zhang, Jiandong Liang, Changyan Wu, Xiao Zou

**Affiliations:** ^1^Institute of Fungus Resources, College of Life Sciences, Guizhou University, Guiyang, China; ^2^Department of Environmental Engineering, Chongqing Vocational College of Resources and Environmental Protection, Chongqing, China; ^3^Basic Medical School, Guizhou University of Traditional Chinese Medicine, Guiyang, China

**Keywords:** blood lead, heavy metal pollution, Alzheimer’s disease, multigenomics, Mendelian randomization

## Abstract

Alzheimer’s disease (AD) is a life-threatening neurodegenerative disease of the elderly. In recent observations, exposure to heavy metals environmental may increase the risk of AD. However, there are few studies on the causal relationship between heavy metal exposure and AD. In this study, we integrated two large-scale summaries of AD genome-wide association study (GWAS) datasets and a blood lead level GWAS dataset and performed the two-sample Mendelian randomization analysis to assess the causality of blood lead level and AD risk. The results showed that there is a significantly positive causality between blood lead level and AD risk both in the inverse-variance weighted (IVW) model and the weighted median estimator (WME) model. An independent additional verification also reached a consistent conclusion. These findings further confirm the conclusions of previous studies and improve the understanding of the relationship between AD pathogenesis and the toxicity of lead in environmental pollution.

## Introduction

Heavy metals are non-biodegradable, and well-documented evidence supports that chronic exposure to heavy metals can cause neurodegenerative diseases (Bush, 2003; Mates et al., 2010). These pollutants arise from rapid urbanization and industrialization, such as municipal waste, traffic, aquaculture, agricultural chemicals, paint coatings, petrochemical industry, electronic industry, mining, and smelting (Tchounwou et al., 2012; Wang et al., 2013; Ojuederie and Babalola, 2017; Fan et al., 2020). Human exposure to heavy metals mainly *via* ingestion of metal-contaminated food, water, and employment in metal-contaminated workplaces (Tchounwou et al., 2012). Several epidemiological studies have shown a significant association between cumulative metals exposure and neurodegenerative diseases (Bjorklund et al., 2018; Bakulski et al., 2020). There is robust evidence that heavy metals can disturb neurotransmitter systems by multiple mechanisms, including the interaction with neurotransmitter receptors, the modification of certain gene and/or protein expression, and the collateral damage of their functions following Reactive Oxygen Species (ROS) production (Bertram and Tanzi, 2005; Carmona et al., 2021). A previous study found that some amyotrophic lateral sclerosis patients have a 2.3- to 3.6-fold increase both in the patellar and tibial lead, which is a dose-dependent increased risk of this disease (Kamel et al., 2002).

There are many kinds of neurodegenerative diseases, including Parkinson’s disease, amyotrophic lateral sclerosis, Lewy body dementia, Alzheimer’s disease (AD), and so on. Among them, AD is the most typical neurodegenerative disease (Bakulski et al., 2020). AD is a neurodegenerative disease that threatens the life of the elderly, and currently, there is no efficient treatment for AD (Bakulski et al., 2020). AD is caused by a variety of environmental, lifestyle, and genetic factors that influence the degeneration of neuronal cells over some time (Bakulski et al., 2020; Huang et al., 2022). The neuropathological features of AD are hyperphosphorylated tau (a microtubule-binding protein), neurofibrillary tangles (NFTs), and aging plaques consisting of accumulated amyloid protein (Aβ) and contained metal ions (Han et al., 2019; Bakulski et al., 2020).

Accumulating evidence suggests that heavy metal pollution may be an important contributor to AD, but there is no comprehensive understanding of the effects of heavy metal pollution on AD. This study attempts to analyze the correlation between heavy metal pollution and AD by the Mendelian randomization. Mendelian randomization analysis is an analytical method for evaluating the observed correlation between a changeable risk or exposure factor and a clinically relevant outcome (Sekula et al., 2016). The use of as many instrumental variables as possible can reduce the concern of weak instrumental bias (Burgess and Thompson, 2011). This research uses genetic variants to assess the causal relationship between heavy metal exposure and AD.

In this study, we first selected the genome-wide association study (GWAS) summary data of AD and environmental pollutants from multiple authoritative databases. Then, we filtered the GWAS summary data and selected independent and matched exposure risk factor-related SNPs as the instrumental variables. Next, based on the instrumental variables with their GWAS summary results, we used two models to assess the causality of environmental pollutants and AD risk by the two-samples Mendelian randomization analysis. Finally, we used three check methods to ensure the reliability of the results of the Mendelian randomization analysis.

## Materials and Methods

### Data Sources

The common water quality pollutants were considered as the exposure risk factors in this study. The related genetic variations of these exposure risk factors were selected by searching the NHGRI-EBI GWAS Catalog^1^ using the keywords: “Cadmium,” “Chromium,” “Mercury,” “Manganese,” “Lead,” “Molybdenum,” and “Nickel.” The NHGRI-EBI GWAS Catalog is a curated collection for delivering the high-quality published (GWAS) summary results of various human traits (Buniello et al., 2019). Finally, we only identified 14 blood lead level-related SNPs from a 5,433-sample size European ancestry GWAS study. This study used the blood samples from the Queensland Institute of Medical Research in Australia and the Avon Longitudinal Study of Parents and Children in the United Kingdom to measure blood lead levels and genotype of the SNPs (Warrington et al., 2015). The details were shown in Supplementary Table 1. The summary of GWAS data on AD is derived from a consortium consisting of the Alzheimer’s Disease Genetics Consortium (ADGC), European Alzheimer’s Disease Initiative (EADI), Cohorts for Heart and Aging Research in Genomic Epidemiology Consortium (CHARGE), and Genetic and Environmental Risk in AD/Defining Genetic, Polygenic and Environmental Risk for Alzheimer’s Disease Consortium (GERAD/PERADES). A total of 10,528,610 variants are genotyped and measured using 21,982 AD individuals and 41,944 controls (Kunkle et al., 2019). In addition, to ensure the reliability of the results, we further used an independent GWAS dataset EFO_0000249, which includes 5,918 AD individuals and 212,874 controls, to conduct a verification using the Mendelian randomization analysis.^2^

### Selection and Filtration of Instrumental Variables

According to the threshold of significant association *P* < 10^–5^, we first selected the 14 blood lead level-related SNPs as the instrumental variables and further discarded the non-biallelic SNPs. Then, we matched the remaining SNPs to the AD GWAS results and attempted to align strands of the palindromic SNPs for allele harmonization. Next, to ensure mutual independence between the instrumental variables, we performed a linkage disequilibrium (LD) analysis and filtered the non-independent SNPs according to the significance threshold, i.e., *r*^2^ < 0.001 within the 10,000 kb window. The samples used to estimate the LD effect were derived from the 1,000 Genome Project European ancestry individuals (Consortium, 2012). Finally, if blood lead level-related SNP is not present in the AD GWAS results, we tried to use the proxy SNPs through LD tagging (*r*^2^ = 1) instead of it and integrated the filtered SNPs with the GWAS results of blood lead level and AD as the instrumental variables.

### Mendelian Randomization Analysis

We used the R package “TwoSampleMR” and its web server “MRBASE” to perform the two-sample Mendelian randomization analysis (Hemani et al., 2018). Particularly, we conducted the inverse-variance weighted (IVW) model and the weighted median estimator (WME) model to assess the causal effect of blood lead level on AD risk. The IVW model ignores the intercept in the regression analysis and uses the inverse of the variance as a weight for the fit. The WME model is a consistency estimator under the assumption that more than half of the instrumental variables are valid. For the IVW model, each inverse-variance was estimated by dividing SNP-AD associations by SNP-blood lead level associations (i.e., Wald ratios). Then, the mean effect of blood lead level on AD risk was estimated by a random effect meta-analysis of the Wald ratios. When the inverse-variance satisfies the primary assumptions of Mendelian randomization analysis [i.e., the inverse-variance: (1) is associated with the exposure, (2) is not associated with the confounders, and (3) does not influence the outcome through some pathways other than the exposure], IVW model can provide accurate estimates (Burgess et al., 2013; Staley and Burgess, 2017). For the WME model, the intercept of the fitted curve was calculated to estimate the average pleiotropy effect across the genetic variants. The WME can also provide a consistent estimate when more than half of the inverse variance satisfies the primary assumptions of Mendelian randomization analysis (Verbanck et al., 2018). The threshold of significant causal effect was set as *P* < 0.05. Moreover, the causal effect was considered positive and negative when the beta value was greater and less than zero, respectively.

### Reliability Check

To ensure the reliability of the results of Mendelian randomization analysis, we performed the horizontal pleiotropy test, heterogeneity test, and sensitivity analysis. Particularly, we used the Egger regression intercept to estimate the magnitude of horizontal pleiotropy. If the SNPs influence the AD risk through a pathway other than the blood lead level, the significant horizontal pleiotropic (*P* < 0.05) can bias the Mendelian randomization estimates (Burgess and Thompson, 2017). Then, we assessed the heterogeneity by a funnel plot. The asymmetry and large spread of the funnel plot indicate a high heterogeneity. The significant threshold was set as *P* < 0.05 (Van Kippersluis and Rietveld, 2018). Finally, we conducted the sensitivity analysis by removing each SNP from the original Mendelian randomization analysis. The leave-one-out sensitivity analysis was used to ascertain if an association is being disproportionately influenced by a single SNP, and the forest plot was used to show the results.

## Results and Discussion

### The Selected Instrumental Variables for Mendelian Randomization Analysis

We collected the summary GWAS data of blood lead levels from the NHGRI-EBI GWAS Catalog, and AD from the EFO_0000249 and a consortium consisting of the ADGC, EADI, CHARGE, and GERAD/PERADES, respectively. All of the samples are derived from European ancestry individuals. The blood lead level GWAS dataset was intersected with two AD GWAS datasets, respectively. After the allele harmonization, LD filtering, and SNP proxy, we selected a total of three SNPs as the instrumental variables for Mendelian randomization analysis which are significantly associated with the blood lead level and independent of each other for the consortium’s AD GWAS dataset. Particularly, SNP rs76153987 (chr3:9173133), rs116864947 (chr7:11666159), and rs6462018 (chr7:27479499) are located in genes SRGAP3, THSD7A, and EVX1, respectively, and all of them are negatively associated with the blood lead level (beta = −0.195, −0.431, and −0.084; *P* = 4 × 10^–6^, 3 × 10^–7^ and 4 × 10^–6^, respectively) (Warrington et al., 2015). The AD GWAS results of them are beta = −0.073, −0.123, and −0.002 and *P* = 0.033, and 0.059 and 0.015, respectively (Table 1). For the EFO_0000249 dataset, we identified two additional SNPs, rs798338 (chr7:78287721 in MAGI2), and rs10121150 (chr9:113369415 in BSPRY), after the screening process. The AD GWAS results of them are beta = −0.015 and 0.069 and *P* = 0.490 and 0.018, respectively (Table 2). The human reference genome hg38 was used in this study. The more detailed information was shown in Supplementary Table 1.

**TABLE 1:** The causality of blood lead level and Alzheimer’s disease (AD) risk by two-sample Mendelian randomization (MR) analysis using the data of AD consortium (Kunkle et al., 2019).

SNP	Position	Effect allele	GWAS of blood lead level		GWAS of AD		Model	MR analysis		Heterogeneity test		Horizontal pleiotropy	
			Beta	*P*-value	Beta	*P*-value		Beta	*P*-value	Cochran’s Q	*P*-value	Intercept	*P*-value
rs76153987	chr3:9214817	T	−0.195	4 × 10^–6^	−0.073	0.0266							
							IVW	0.245	0.0103	2.161	0.34		
rs116864947	chr7:11705786	T	−0.431	3 × 10^–7^	−0.123	0.0376						−0.029	0.44
							WME	0.262	0.0367	0.684	0.41		
rs6462018	chr7:27519118	G	−0.084	4 × 10^–6^	−0.002	0.8994							

**TABLE 2:** The causality of blood lead level and AD risk by two-sample MR analysis using the data of EFO_0000249.

SNP	Position	Effect allele	GWAS of blood lead level		GWAS of AD		Model	MR analysis		Heterogeneity test		Horizontal pleiotropy	
			Beta	*P*-value	Beta	*P*-value		Beta	*P*-value	Cochran’s Q	*P*-value	Intercept	*P*-value
rs76153987	chr3:9214817	T	−0.195	4 × 10^–6^	−0.036	0.4025							
							IVW	0.242	0.0046	3.297	0.51		
rs116864947	chr7:11705786	T	−0.431	3 × 10^–7^	0.128	0.0333							
rs6462018	chr7:27519118	G	−0.084	4 × 10^–6^	−0.005	0.8136						–0.026	0.385
rs798338	chr7:78287721	A	−0.111	4 × 10^–6^	−0.015	0.4901							
							WME	0.220	0.0059	2.269	0.52		
rs10121150	chr9:113369415	C	−0.143	3 × 10^–8^	0.069	0.0183							

### The Causality of Blood Lead Level and Alzheimer’s Disease Risk

**FIGURE 1:**
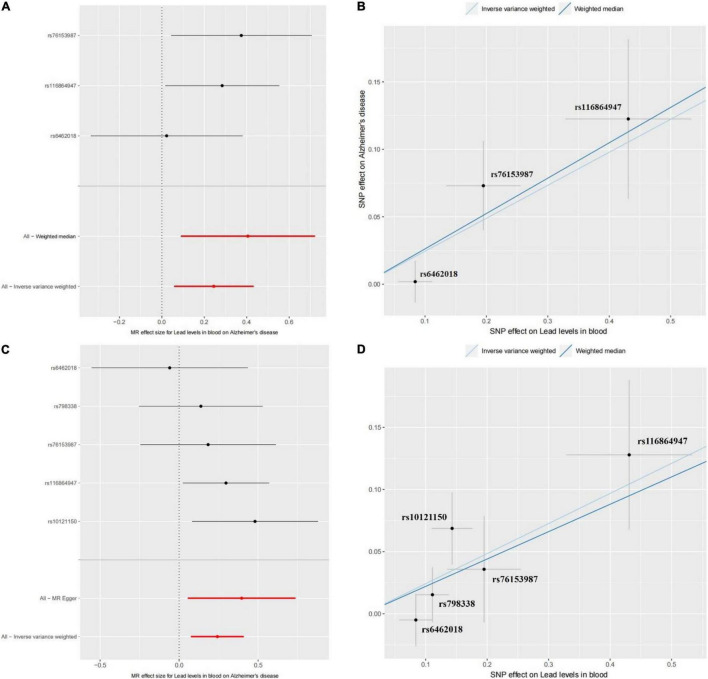
The Mendelian randomization (MR) analysis for the causality of blood lead level and Alzheimer’s disease (AD) risk. **(A)** The forest plot represents the causal effect of blood lead level on AD using the Wald ratio. The Mendelian randomization using singly each SNP and all SNPs by the WME and IVW models are shown in it. **(B)** The method comparison plot shows the SNP effects on AD against SNP effects on blood lead levels in the WME and IVW models. Each method has a different line, and the slope of the line represents the causal association. Panels **(C,D)** show the forest plot of causal effect and the method comparison plot of WME and IVW models for the EFO_0000249 dataset, respectively.

Using the three SNPs with their GWAS results about blood lead level and AD, we performed the two-sample Mendelian randomization analysis to assess the causal effect of blood lead level on AD risk. The results of the IVW model showed that there is a significant positive causality between blood lead level and AD risk (beta = 0.2445 and *P* = 0.0103). The whole confidence interval of Mendelian randomization effect size for blood lead level on AD is greater than zero (Figure 1A). The WME model showed similar results (beta = 0.2621 and *P* = 0.0382) (Figure 1A). As Figure 1B shows, the influence of the three SNPs on blood lead level and AD in the two models exhibits good consistency. To ensure the reliability of the results, we further performed a Mendelian randomization analysis using the five SNPs from the EFO_0000249 dataset. We found a similar result, i.e., beta = 0.2421 and 0.2203 and *P* = 0.0046 and 0.0059 in IVW and WME model, respectively (Figure 1C). The influence of the five SNPs on blood lead level and AD in the two models also exhibits a good consistency (Figure 1D). These results suggest that the elevated blood lead level increases the risk of AD. The previous studies reported that the toxicity of lead gives rise to severe environmental pollution with the use of petrol and its exposure results in cognitive decline in elderly men and women. Moreover, the blood lead level was found significantly higher in the patients with AD and is associated with an increase in AD mortality after adjusting for identified confounders (Laidlaw et al., 2017; Fathabadi et al., 2018; Horton et al., 2019). Our findings are consistent with these studies and further confirm previous conclusions, which suggest that the exposure of lead may damage the nervous system and increase risk of AD.

### Reliability Check

**FIGURE 2:**
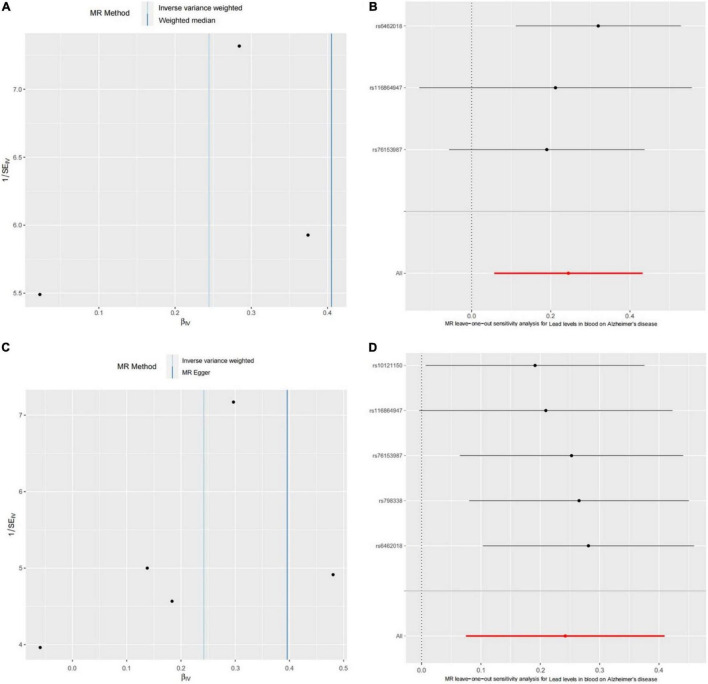
The heterogeneity test and sensitivity analysis of the Mendelian randomization analysis. **(A)** Funnel plot to assess heterogeneity. Asymmetry and large spread suggest a high heterogeneity. **(B)** The forest plot of leave-one-out sensitivity analysis shows if an association is being disproportionately influenced by a single SNP. Each black point represents the Mendelian randomization analysis excluding that particular SNP. Panels **(C,D)** show the results of the heterogeneity test and leave-one-out sensitivity analysis for the EFO_0000249 dataset, respectively.

We further performed the horizontal pleiotropy test, heterogeneity test, and sensitivity analysis to check reliability of the Mendelian randomization analysis. For the consortium’s AD GWAS dataset, the results showed that there is no directional horizontal pleiotropy affecting the IVW and WME estimate (intercept = −0.029 and *P* = 0.438) (Table 1). Then, Cochran’s Q test showed that there is also no significant heterogeneity in IVW (Cochran’s Q-statistic = 2.161 and *P* = 0.340) and WME estimate (Cochran’s Q-statistic = 0.684 and *P* = 0.408) (Figure 2A). Moreover, the leave-one-out sensitivity analysis showed that the results of the Mendelian randomization analysis do not extremely change when we removed each of the SNP orderly (Figure 2B). For the EFO_0000249 dataset, the similar results also showed a non-directional horizontal pleiotropy (intercept = −0.026 and *P* = 0.385) (Table 2), non-significant heterogeneity in IVW (Cochran’s Q-statistic = 3.297 and *P* = 0.510), WME estimate (Cochran’s Q-statistic = 2.269 and *P* = 0.520) (Figure 2C), and insignificant changes in sensitivity analysis (Figure 2D). These results demonstrate that the causality of blood lead level and AD is reliable, and further suggest that the elevated blood lead level increases the risk of AD.

## Conclusion

The lead pollution is a serious environmental problem and damages to the human central nervous system. In this study, we integrated two large-scale summary AD GWAS datasets and a blood lead level GWAS dataset to assess the causality of blood lead level and AD risk by the two-sample Mendelian randomization analysis. After the reliability check, we found a significant positive causality between blood lead level and AD risk. Our findings suggest that the exposure of lead may increase risk of AD, which is further confirm the results of previous studies and benefit to understanding of AD pathogenesis and the toxicity of lead in environmental pollution.

## Data Availability Statement

The original contributions presented in this study are included in the article/Supplementary Material, further inquiries can be directed to the corresponding author.

## Author Contributions

XZ and CL designed and performed the research, analyzed the data, and wrote the manuscript. XZ, CL, YZ, JL, and CW collected the data. XZ reviewed and modified the manuscript. All authors discussed the results and contributed to the final manuscript.

## Conflict of Interest

The authors declare that the research was conducted in the absence of any commercial or financial relationships that could be construed as a potential conflict of interest.

## Publisher’s Note

All claims expressed in this article are solely those of the authors and do not necessarily represent those of their affiliated organizations, or those of the publisher, the editors and the reviewers. Any product that may be evaluated in this article, or claim that may be made by its manufacturer, is not guaranteed or endorsed by the publisher.

## Footnotes

1
https://www.ebi.ac.uk/gwas

2
https://r5.risteys.finngen.fi/phenocode/G6_AD_WIDE
